# Loading Rate in Single-Leg Drop Landing: Associations With Risk Factors for Ankle Sprain

**DOI:** 10.7759/cureus.88417

**Published:** 2025-07-21

**Authors:** Yasushi Kurihara, Hironori Ohsugi, Yutaka Kuwae, Taizan Fukaya, Daigo Fujikawa

**Affiliations:** 1 Department of Physical Therapy, Faculty of Social Work Studies, Josai International University, Togane, JPN; 2 Home Medical Care, Keisei Koiwa Home Medical Clinic, Edogawa-ku, JPN

**Keywords:** ankle sprain, loading rate, lower limb joint angle, peroneus longus parameter, single-leg drop landing

## Abstract

Aim*:* The aim of this study is to investigate the association between the loading rate (LR) during single-leg drop landing (SDL) and biomechanical factors contributing to ankle sprains (AS).

Methods: Seventeen healthy young men (mean age: 19.8 ± 0.8 years) were included in this study. A three-dimensional motion analyzer and a floor reaction force sensor were used to analyze SDLs conducted from a 20-cm-high platform onto a floor positioned 30 cm away. The change in lower limb joint angles from landing to the point of maximum vertical floor reaction force was calculated. Additionally, peroneus longus parameters, including maximum muscle tension and timing, were measured using musculoskeletal modeling software. Associations of the LR with changes in lower limb joint angles and peroneus longus parameters were analyzed using correlation analysis (p<0.05).

Results: The LR was negatively correlated with the hip flexion, knee flexion, and ankle dorsiflexion angles (r = -0.781, -0.649, and -0.489; p = 0.0002, 0.005, and 0.047, respectively). In contrast, the LR was positively correlated with the timing of the peroneus longus muscle (r = 0.672, p = 0.003). No other correlations were observed.

Conclusions: A higher LR during SDL was associated with reduced sagittal plane joint motion and delayed peroneus longus activation, both of which are biomechanical factors linked to AS onset. These findings suggest that the LR may serve as a useful marker for identifying individuals at elevated risk of AS.

## Introduction

Ankle sprains (AS) account for up to 30% of all sports-related injuries, making them the most frequent musculoskeletal injuries of the lower extremity in both athletes and the general population [1]. Most AS cases are inversion sprains, and the mechanisms of injury are classified into contact and non-contact injuries, such as those caused by jump landings and turning motions [2,3]. The high incidence of AS is partly attributed to frequent reinjury. Attenborough et al. [4] demonstrated that 46% of AS cases in volleyball and 28% of AS cases in basketball were reinjuries. Additionally, a history of AS is the strongest risk factor for future injuries, with several people with many injured individuals experiencing multiple episodes of AS [5]. Among lower-extremity musculoskeletal injuries, AS have the highest recurrence rate [6] and result in chronic ankle instability (CAI), characterized by tissue injury and dysfunction around the ankle joint. This high recurrence rate is attributed to inadequate rehabilitation and premature return to competition [7,8], thereby limiting sports performance [9]. Therefore, providing appropriate post-traumatic therapy after AS onset and establishing clear indicators for return to competition are central to decreasing recurrence rates [10]. The National Athletic Trainers' Association's Position Statement [10] recommends using athletes' self-assessments of subjective function [11] and the Star Excursion Balance Test [12] as indicators to return to competition. These indirect assessments do not directly measure mechanical loading during jump landings [2,3], which causes several injuries. The floor reaction force analysis facilitates measuring mechanical loading during landing. Simpson et al. [13] demonstrated that people with CAI exhibit higher maximum vertical floor reaction force during single-leg drop landings (SDLs) than controls. Furthermore, the loading rate (LR) represents the rate at which force is applied to the body during landing. It is calculated by dividing the peak vertical ground reaction force by the time taken to reach that peak from initial foot contact [14]. A high LR implies poor shock attenuation and a high mechanical load in a short period, indicating that individuals with CAI perform landing movements with high mechanical loading. Although a previous study [15] mentions the relationship between the LR and the risk of ACL injury, it does not provide a sufficient conclusion regarding the prediction of AS onset, nor does it clarify which biomechanical characteristics the LR may reflect.

This study aimed to examine the relationship between the LR during SDL and biomechanical factors linked to AS risk, with the hypothesis that an elevated LR is associated with deficits commonly observed in individuals with CAI [16-18]. We will first obtain the data of LR in healthy individuals without a history of AS and use them as the fundamental data to utilize the LR as a novel evaluation index for future risk assessment and return to competition decision processes.

## Materials and methods

Participants

This study included 17 healthy young men. Individuals with a history of orthopedic surgery or diseases involving the lower extremities, including AS, within the past six months were excluded. All participants were informed both verbally and in writing about their rights, their freedom to participate, their ability to withdraw from the study, the research content, and potential physical effects, per the tenets of the Declaration of Helsinki. Informed consent was obtained from all participants. This study was approved by the Josai International University Committee for Medical and Life Science Research on Human Subjects (approval number: 25N230016).

SDL

Participants conducted the SDL task while standing with their upper limbs crossed in front of their chest. They were instructed to land on a floor reaction force gauge positioned 30 cm in front of a 20-cm-high platform. The dominant leg was defined as the leg used for kicking the ball, whereas the non-dominant leg was defined as the landing leg. Niu et al. [19] reported that the non-dominant leg has different joint motion and loading characteristics during landing, suggesting an increased risk of injury. Therefore, the non-dominant leg was selected for analysis in this study as the side more suitable for elucidating the mechanism of AS. After landing, they were instructed to remain stationary for 3 s. Successful trials were defined as those in which the participants remained still after landing. All participants were provided with sufficient practice time to become fully familiar with the task. Measurements were repeated until each participant successfully completed one valid trial. All participants achieved a successful trial within three attempts, and no signs of fatigue or compensatory movements were observed.

Biomechanics

 A three-dimensional motion analyzer (KSTRL-RT-10, Cortex6, 10 cameras, 200 Hz, Mac3D) and a floor reaction force sensor (TF-4060 1000 Hz, Toshiba Tec. Corp., Tokyo, Japan) were used for the biomechanical assessment. Reflective markers, measuring 14 mm in diameter, were affixed at 29 anatomical points throughout the body per the Hayes marker set protocol (the top, front, and back of the head, the right scapula, sacrum, and bilaterally on segments of the shoulders, elbows, wrists, anterior superior iliac spine, lateral thighs, medial and lateral knees, lateral shank, medial and lateral malleoli, heels, and toes). The obtained data were used to calculate the lower limb joint angles on the sagittal and frontal planes of the non-dominant leg and the vertical floor reaction force using Visual3D (C-Motion Inc., MD, USA). The hip, knee, and ankle joint angles during the maximum vertical floor reaction force were extracted, and changes in each joint angle after floor contact were determined. Additionally, parameters of the peroneus longus muscle, which is associated with AS onset [18,20], were analyzed using the musculoskeletal modeling software nMotion Musculous (NAC Inc., Tokyo, Japan) [21,22]. Based on the Genetic Hayes marker set, which is based on reflex markers across 29 anatomical points, the maximum tension and timing of the peroneus longus muscle contraction in the non-dominant leg side were calculated after floor contact.

The LR was calculated as the peak vertical ground reaction force (normalized to body weight) divided by the time from initial contact (defined as GRF >10 N) to peak force [14]. Higher LR values were associated with poorer impact attenuation and greater mechanical load over a shorter period.

Statistical analysis

Statistical analyses were conducted using IBM SPSS Statistics for Windows, Version 27 (Released 2020; IBM Corp., Armonk, New York, United States). The association of the LR and lower-extremity joint angle changes (hip, knee, and ankle joints) and peroneus longus muscle parameters (maximum tension value and timing) was analyzed during SDL of the non-dominant leg. The normality of the data distribution was assessed using the Shapiro-Wilk test. Depending on the distribution, either Pearson’s or Spearman’s correlation coefficient was calculated. A significance level of 5% was applied for all statistical analyses.

## Results

Table 1 summarizes the background characteristics of all participants. Additionally, Table 2 presents the LR, lower-extremity joint angle changes, and peroneus longus muscle parameters (maximum tension value and timing) derived from the SDL. Table 3 summarizes the correlations between the LR and each parameter. Higher LRs were associated with reduced hip, knee, and ankle joint flexion, suggesting stiffer landings with limited joint absorption (r = -0.781, -0.649, and -0.489; p = 0.0002, 0.005, and 0.047, respectively). Additionally, delayed peroneus longus activation was linked to higher LR, indicating potentially compromised lateral ankle stabilization (r=0.672, p=0.003). No other associations were observed.

**Table 1:** Participant characteristics Data are expressed as mean ± standard deviation. Medical history: Participants with a history of orthopedic surgery or orthopedic disease of the lower extremities within the past six months.

	N=17	
Age (years)	19.8±0.8	
Height (cm)	172.1±5.0	
Weight (kg)	64.9±10.8	
Body mass index (BMI)	21.8±2.8	
Medical history	none	

**Table 2:** Biomechanics data N = 17. Data are expressed as mean ± standard deviation. Loading rate; peak value of the vertical floor reaction force divided by the time from foot contact to the peak value ROM: range of motion; change in the joint angle from the time of foot contact to the point at which the maximum vertical floor reaction force is reached. Maximum muscle tension of peroneus longus: maximal muscle tension of peroneus longus muscle after foot ground contact.

Item		Value	
Loading rate (N/w/ms)		0.05±0.02	
Hip flexion-extension ROM (°)		2.7±3.4	
Hip abduction-adduction ROM (°)		1.4±1.5	
Knee flexion-extension ROM (°)		32.0±7.3	
Knee abduction-adduction ROM (°)		2.3±5.2	
Ankle flexion-extension ROM (°)		17.7±5.1	
Ankle inversion-eversion ROM (°)		17.3±16.2	
Maximum muscle tension of peroneus longus (N/w)		4.5±2.6	
Timing of maximum muscle tension of peroneus longus (ms)		94.7±45.1	

**Table 3:** Correlation coefficients between the loading rate and biomechanics data (r) N = 17, *: p<0.05, and **: p<0.01 LR: loading rate; ROM: range of motion

			LR (N/w/ms)	
			r	p-value
Hip flexion-extension ROM (°)			-0.781**	0.0002
Hip abduction-adduction ROM (°)			-0.093	0.722
Knee flexion-extension ROM (°)			-0.649**	0.005
Knee abduction-adduction ROM (°)			-0.284	0.268
Ankle flexion-extension ROM (°)			-0.489*	0.047
Ankle inversion-eversion ROM (°)			-0.086	0.743
Maximum muscle tension of peroneus longus (N/w)			0.007	0.978
Timing of maximum muscle tension of peroneus longus (ms)			0.672**	0.003

## Discussion

This study confirmed our hypothesis that higher LRs during SDL are associated with biomechanical factors implicated in AS onset.

Association between the LR and joint angle changes

The LR was negatively correlated with changes in the ankle dorsiflexion angle. Self et al. [20] and Martinez et al. [23] mechanistically analyzed SDL and reported an association between a landing strategy characterized by high ankle plantar flexion angles at ground contact and lower floor reaction force. Similarly, our results demonstrated a comparable trend. The ankle joint is central to shock absorption during one-leg landing, particularly via the triceps muscles [24]. Limited dorsiflexion following ground contact may reflect poor shock absorption, leading to higher mechanical loading during SDL. People with a history of AS and CAI experience lower ankle joint plantar flexion angles immediately before SDL and ankle joint dorsiflexion angles after landing than controls [25-27]. Thus, ankle dorsiflexion angle changes after landing may contribute to AS onset. Therefore, our results demonstrate an association between LR and ankle dorsiflexion angle change, suggesting the application of LR as an evaluation index for assessing the risk factors for AS onset.

Except for ankle dorsiflexion angle changes, the LR was negatively correlated with changes in knee and hip flexion angles. Controlling the center of gravity during landing involves not only the ankle joint but also the kinetic chain of lower limbs, including the knee and hip joints [28]. Furthermore, the shock-absorbing function during landing increases when the hip and knee flexion angles are higher after landing [29]. In contrast, the LR tends to be higher for lesser changes in knee joint and hip flexion angles after landing, supporting our results.

Association between the LR and peroneus longus parameters

The LR was positively associated with peroneus longus parameters (maximum tension value and timing) in this study. A high LR delayed the timing of achieving maximum tension by the peroneus longus muscle after landing. The peroneus longus muscle exerts an eversion effect on the ankle joint and contributes to its dynamic stabilization [30]. Delayed peroneal response may predispose to inversion injury due to lack of timely eversion torque. Nonetheless, after AS, its function declines and morphological changes occur, which may contribute to recurrent sprains [31]. Multiple AS events have been associated with decreased strength in the ankle joint extrinsic muscles, including the peroneal muscle group [32]. Additionally, Hoch et al. [33] identified a neuromuscular control problem where the reaction time of the peroneus longus muscle was delayed in patients with a history of CAI compared with that in controls. Therefore, delayed reaction time may impair the ability to stabilize and protect the ankle joint from sudden inversion [34,35], thereby increasing mechanical load during landing. Overall, our findings are congruent with those of previous studies.

The LR was not associated with maximal peroneus longus tension. Arnold et al. [32] reported decreased ankle eversion muscle strength in patients with a history of CAI. However, this finding was obtained by measuring concentric contraction; therefore, they may differ from our results. Previous studies using electromyography have reported lower peroneus longus activity before landing during SDL in patients with a history of CAI than in controls [17,18]. This necessitates re-examining the association between the LR and peroneus longus muscle activities based on the method used to measure muscle activity.

This study has a few limitations. First, it only demonstrated an association between the LR and the factors contributing to AS onset, and a causal association could not be determined. Therefore, a longitudinal study is required to verify this causal association. Second, the study was conducted on young, healthy adults and did not examine differences in sex, age, or sports characteristics. AS incidence may vary with age and athletic events [36,37]. Therefore, the applicability of our results to childhood and adolescence, characterized by a developing musculoskeletal system, remains unclear, particularly for first-ever sprains and in different sports. Third, although the original power analysis indicated that a sample size of 29 participants was required, data collection was completed with 17 participants due to recruitment limitations and practical constraints during the study period. Despite the smaller sample size compared to the initial plan, the primary trends were clearly observed, and the results are considered to have a certain level of statistical interpretability. Lastly, it should be noted that this study was conducted based on a single trial for each participant. Therefore, the reproducibility and reliability of the SDL task were not examined. Thus, future studies should include athletes of different ages and sports and assess muscle activation patterns longitudinally to clarify whether a high LR can prospectively predict AS risk.

## Conclusions

The LR during SDL was associated with changes in ankle joint dorsiflexion angles immediately after landing as well as with the timing of reaching the maximum tension of the peroneus longus muscle, supporting our hypothesis. These results indicate that the LR may have the potential to predict the risk of AS occurrence and serve as a novel evaluation index for return to sport decisions following AS. Further studies are warranted to determine whether the LR improves AS recurrence rates.
